# Supplementary material for: Glycan Epitopes on 201B7 Human-Induced Pluripotent Stem Cells Using R-10G and R-17F Marker Antibodies
Source: Biomolecules. 2021 Mar 29;11(4):508. doi: 10.3390/biom11040508 (PMC8065539; doi:10.3390/biom11040508)

biomolecules

MDPI

Figure S1. Elution profile obtained by first affinity chromatography of 201B7 cell crude extract using an R-10G column followed by Western blotting using R-10G.

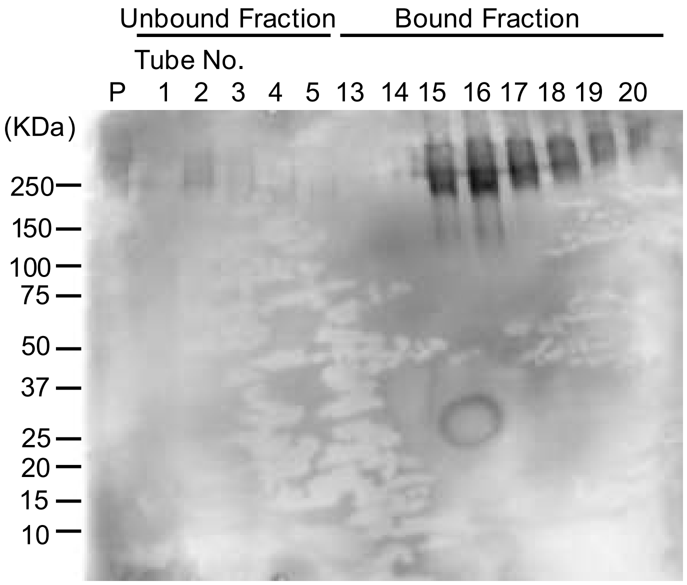

Figure S2. Elution profile obtained by re-affinity chromatography of the pooled bound fraction in Figure S1 using an R-10G column followed by Western blotting using R-10G.

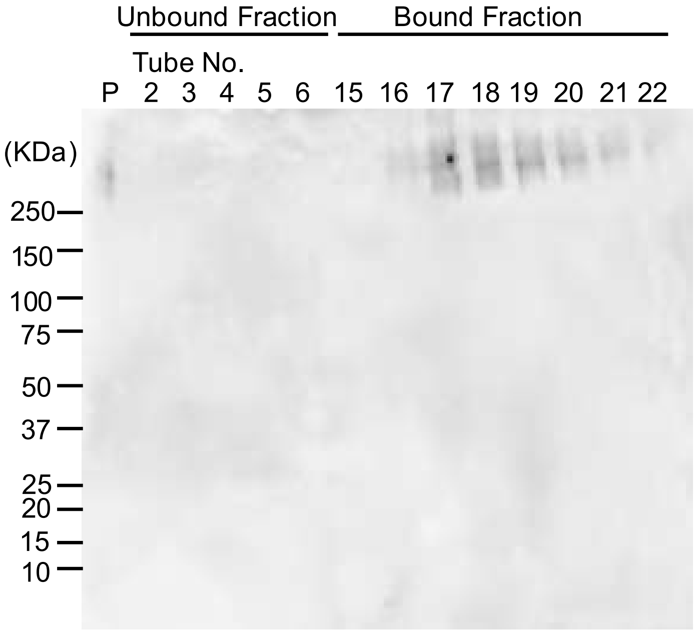

Figure S3. Semi-quantitative comparison of the expression level of the R-10G epitope on 201B7 and Tic cells by Western blotting.

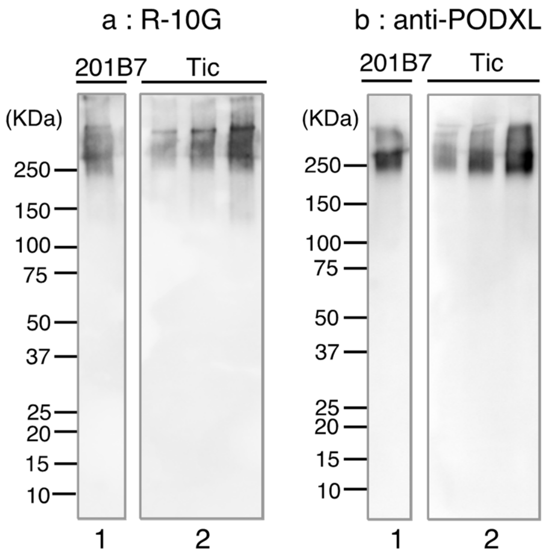

Figure S4. Localization of G-10G (a) and R-17F(b) epitopes on cultured 201B7 cells visualized on laser confocal microscopy.

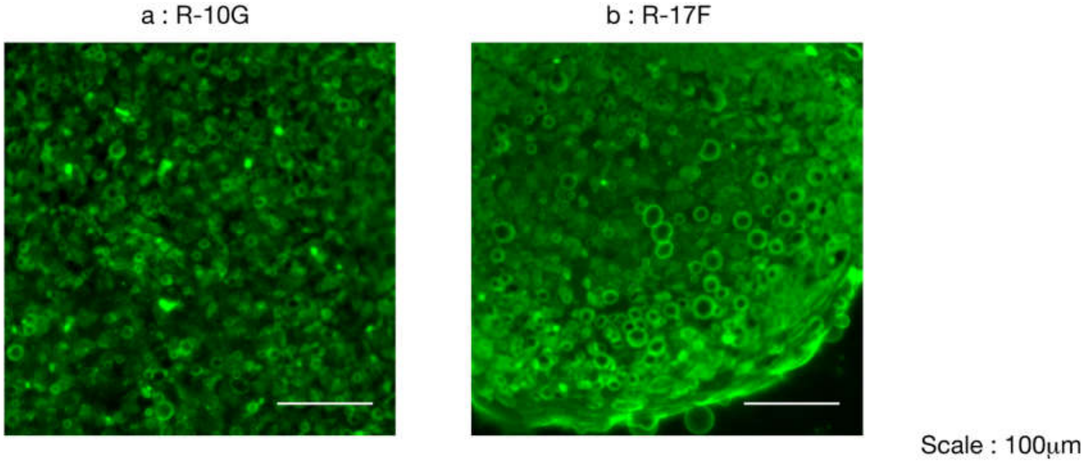

Supplement: Supplementary file 1 [file biomolecules-11-00508-s001.zip › biomolecules-1155208-supplementary.pdf]
